# Cellular and molecular actors of myeloid cell fusion: podosomes and tunneling nanotubes call the tune

**DOI:** 10.1007/s00018-021-03875-x

**Published:** 2021-07-23

**Authors:** Ophélie Dufrançais, Rémi Mascarau, Renaud Poincloux, Isabelle Maridonneau-Parini, Brigitte Raynaud-Messina, Christel Vérollet

**Affiliations:** 1 Institut de Pharmacologie et Biologie Structurale, IPBS, Université de Toulouse, CNRS, UPS, Toulouse, France; 2 International Associated Laboratory (LIA) CNRS “IM-TB/HIV” (1167), Toulouse, France; 3 International Associated Laboratory (LIA) CNRS “IM-TB/HIV” (1167), Buenos Aires, Argentina

**Keywords:** Cell-to-cell fusion, Adhesion, Osteoclasts (OCs), Multinucleated giant cells (MGCs), Podosomes, Tunneling nanotubes (TNTs)

## Abstract

Different types of multinucleated giant cells (MGCs) of myeloid origin have been described; osteoclasts are the most extensively studied because of their importance in bone homeostasis. MGCs are formed by cell-to-cell fusion, and most types have been observed in pathological conditions, especially in infectious and non-infectious chronic inflammatory contexts. The precise role of the different MGCs and the mechanisms that govern their formation remain poorly understood, likely due to their heterogeneity. First, we will introduce the main populations of MGCs derived from the monocyte/macrophage lineage. We will then discuss the known molecular actors mediating the early stages of fusion, focusing on cell-surface receptors involved in the cell-to-cell adhesion steps that ultimately lead to multinucleation. Given that cell-to-cell fusion is a complex and well-coordinated process, we will also describe what is currently known about the evolution of F-actin-based structures involved in macrophage fusion, i.e., podosomes, zipper-like structures, and tunneling nanotubes (TNT). Finally, the localization and potential role of the key fusion mediators related to the formation of these F-actin structures will be discussed. This review intends to present the current status of knowledge of the molecular and cellular mechanisms supporting multinucleation of myeloid cells, highlighting the gaps still existing, and contributing to the proposition of potential disease-specific MGC markers and/or therapeutic targets.

## Introduction

The capacity of cells to fuse and form syncytia or multinucleated cells is evolutionarily conserved among eukaryotes. Cell-to-cell fusion is an essential process during fertilization (fusion of sperm and egg), formation of the placenta (fusion of trophoblast cells), and formation of skeletal muscle (fusion of myoblasts into myotubes) [1]. One of the cell types that can undergo cell-to-cell fusion is the macrophage, which has high fusogenic properties. Indeed, macrophages have the ability to fuse under both physiological and pathological conditions, leading to the formation of multinucleated giant cells (MGCs). The most intensively studied MGCs derived from macrophages are osteoclasts (OCs), which are the exclusive bone-resorbing cells essential for bone homeostasis. Many other MGC subtypes have been described in pathological lesions, especially in infectious and non-infectious chronic inflammatory conditions. These different types of MGCs share common mechanisms of formation but also display unique properties [2, 3] that will be detailed in this review. However, MGCs remain difficult to characterize and classify because their phenotypes vary depending on their environment, the nature of the fusogenic stimuli [4–6], and their function [2, 7].

The formation of MGCs by myeloid cell fusion is a multi-step process that is spatio-temporally regulated. First, cells need to acquire fusion competence, which is under the control of both exogenous stimuli and endogenous signaling pathways. This activation then triggers adhesion of the fusion-competent cells to a permissive substrate, cell motility, cell-to-cell interactions, and finally membrane fusion [3]. Here, we will describe the cellular structures and the molecular mechanisms used by myeloid cells to adhere and fuse with each other or with other cell types. In particular, we will review the diversity of the F-actin-based structures involved in macrophage fusion: podosomes, podosome-related zipper-like structures, and tunneling nanotubes (TNTs). In this context, the potential localization and role of the main molecular actors already described in the fusion process will be discussed.

## Different types of multinucleated giant cells from the monocytic lineage

In addition to bone-resorbing OCs, there are different types of MGCs with a monocyte/macrophage origin. We decided here to focus on the three best-characterized MGCs to date: the foreign body giant cells (FBGCs) formed in response to macroscopicorganic and inorganic materials, Langhans giant cells, generated in response to microbial infection, and MGCs induced by HIV-1 infection. There is also evidence suggesting that macrophages might fuse with non-myeloid cells, such as somatic cells [8], tumor cells [9, 10] or HIV-1-infected T lymphocytes [11, 12]. For clarification, we will use the term ‘homotypic’ when fusion occurs between cells of the same cell type (e.g. between two macrophages or two OC precursors) and ‘heterotypic’ when fusion occurs between different cell types. To account for the complexity of the fusion processes, we will also discuss the possibility of fusion between cells having a common origin but being at different stages of differentiation, in particular in the case of fusion of OC precursors [13, 14].

### Homotypic fusion

OCs are the unique type of myeloid-derived MGCs that form under physiological conditions. They are the exclusive bone-resorbing cells and, together with bone-synthesizing osteoblasts and osteocytes, they constitute the major actors in bone remodeling. OCs originate from the fusion of monocytic precursors, mainly under the control of Macrophage Colony-Stimulating Factor (M-CSF) and Receptor Activator of Nuclear Factor-κB Ligand (RANK-L) [15–17]. RANK-L binding to its receptor leads to the activation of the master transcription regulator of osteoclastogenesis, the Nuclear Factor of Activated T Cells 1 (NFATc1) and the increased expression of resorption-related genes [18]. OC attachment to bone is mediated by a specific structure, the sealing zone, which is composed of a dense array of inter-connected F-actin structures called podosomes [16, 19–21] (see “Role of podosomes and zipper-like structures in myeloid cell fusion” below). The sealing zone participates in the creation of a confined resorption environment, where protons and osteolytic enzymes are secreted [19]. Recent studies in vivo show that mature OCs are most often formed by sequential fusion events with mononucleated OC precursors, suggesting the addition of one nucleus at a time [22, 23]. This progressive process involves fusions between heterogeneous myeloid precursors, whose phenotype could evolve according to the number of nuclei [13, 24, 25]. The formation and function of OCs are tightly controlled in vivo, since dysregulation of OC differentiation and/or function may lead to bone defects, such as osteopetrosis or osteoporosis. Although the specific role of multinucleation in the osteolytic process remains unclear, it has been proposed that cell-to-cell fusion allows OCs to cover a larger bone area to enhance bone resorption activity. In support of this hypothesis, mononuclear or poorly fused OCs degrade bone tissue less efficiently than giant and multinucleated OCs [26–29]. On the other hand, in some pathological conditions such as Paget's disease, bone fragility observed in patients are associated with a strong increase in the number of nuclei per OC but also in OC number and responsiveness to osteoclastogenic signals, among other modified parameters [30]. In addition, infection of OCs with several pathogens (e.g. *Staphylococcus aureus* and HIV-1) leads to an increase in their ability to fuse and degrade the bone matrix [31–34]. However, the functionality of some proteins involved in OC fusion in vitro does not systematically correlate with altered OC differentiation or bone phenotype in vivo, at least under physiological conditions [35, 36]. These discrepancies between in vitro and in vivo observations will be discussed throughout this review.

FBGCs commonly form at the tissue/material interface of implanted medical biomaterials or in tissues where foreign particles or organisms are too large to be phagocytosed [4, 7]. In response to these exogenous materials, acute and chronic inflammation occurs in a sequential fashion, leading to a local increase of interleukin-4 (IL-4) and interleukin-13 (IL-13). In vitro, IL-4- or IL-13- induced FBGC-like cells may have up to one hundred nuclei dispersed throughout the cytoplasm [37]. These MGCs exhibit specific cytokine secretion profiles and maintain some macrophage surface expression markers, whereas the CD14 monocyte marker is down-modulated, resulting in giant cells with a phenotype distinguishable from that of unfused macrophages and other MGCs [38–41]. This phenotype is dependent on material surface chemistry [42]. Although the exact role of FBGCs remains unclear, they are able to phagocytise large and complement-opsonized materials more efficiently than their unfused precursors, and their formation in vivo accompanies the elimination of complement-amyloid deposits [43, 44], suggesting that MGCs are more than the sum of their mononucleated macrophage counterparts.

MGCs are also associated with pathological contexts such as lesions of Langerhans cell histiocytosis [45] and granuloma disorders including sarcoidosis [46], helminthic schistosomiasis [47], and microbial infections. The first granuloma was described by Langhans in the lungs in response to infection by *Mycobacterium tuberculosis* (Mtb), the primary causative agent of tuberculosis [2]. In the early stages of infection, the granuloma consists of a compact and organized aggregate of epithelioid cells (highly specialized, differentiated macrophages) surrounded by a ring of lymphocytes. At later stages, the granuloma develops a fibrous capsid that isolates the core of infected macrophages, reduces vascularization, and thus limits bacilli spread. The plasticity of macrophages is essential for granuloma maturation and dynamics. In particular, macrophages can form MGCs or differentiate into foam cells, characterized by an accumulation of lipids [48]. It is generally accepted that these MGCs, called Langhans giant cells, result from cell-to-cell fusion under the control of inflammatory cytokines. However, it has been proposed that they can also result from defects in cell division [6, 49, 50]. In vitro, the combination of GM-CSF exposition with IFN-γ or IL-3 is sufficient to induce the formation of Langhans giant-like cells with approximately 15 nuclei arranged in a circular pattern [37]. Moreover, within a human in vitro model of granuloma, the fusion of MGCs can be triggered by mycobacterial envelope glycolipids [51]. It remains unresolved whether these MGCs are beneficial or detrimental to the host, as granuloma aggregates restrain Mtb dissemination but do not eliminate all bacilli, promoting their persistence [52]. Several studies have made it possible to decipher their dual roles. In granuloma models, infection with a virulent strain of Mtb induces large MGCs that can no longer mediate bacterial uptake, whereas infection with less virulent species results in MGCs of smaller size but retaining phagocytic capabilities [53]. Moreover, following infection with Mtb, macrophages produce high levels of nitric oxide that drive the transformation of macrophages into giant cells permissive for bacilli persistence [54]. On the other hand, MGCs in tuberculous lymph-nodes highly express extracellular matrix-degrading enzymes, which may promote tissue damage [55]. It is clear that MGCs play a central role in the maintenance of chronic infection and associated tissue damage, however, their role during Mtb infection needs to be further clarified.

Virus-induced fusion of macrophages and more generally of myeloid cells, to our knowledge, has only been studied in the context of HIV-1 infection. Membrane fusion is a mechanism commonly used by several families of enveloped viruses (e.g., Herpesviridae, Paramyxoviridae, Flaviridae, Retroviridae or Coronaviridae) to enter target cells. This process is mediated by fusogenic proteins of the viral envelopes. During productive infection, the host cell expresses new viral envelope proteins at its plasma membrane, which are able to bind to their receptors on neighboring cells, leading to cell-to-cell fusion and thus virus-induced syncytium formation [56–58]. Myeloid cells, particularly macrophages, are an important target for HIV-1, and HIV-1-induced MGC formation is considered a hallmark of macrophage infection. The fusion mechanism is dependent on the interaction between the viral protein gp120 and its receptor CD4 [59]. It can also be supported by another viral protein, Nef, which modulates the organization of the F-actin cytoskeleton of macrophages (i.e. podosomes) favoring macrophage fusion [60, 61]. Importantly, this phenomenon, which is observed in vitro, is relevant in vivo*,* as many reports have shown the presence of HIV-positive MGCs in several tissues from infected patients, notably in secondary lymphoid organs [62], gut-associated lymphatic tissue [63], and brain [64]. Further histological analyses confirmed the myeloid origin of these MGCs. OCs have also been identified as cell targets for HIV-1, and their multinucleation and function are exacerbated after infection [31, 32]. HIV-1-induced MGCs are highly virus productive, present strong differences in their cytokine profiles, and have exacerbated migration capabilities, which likely contribute to viral dissemination in many host tissues [11, 12, 60, 65–67]. They have also been proposed to play a role in viral persistence by acting as virus reservoirs since they are long-lived cells that are resistant to virus-induced cytotoxicity, cell host restriction, CD8^+^ T lymphocyte-mediated killing, and some antiretroviral therapies [68–73].

### Heterotypic fusion

Myeloid cells are able to fuse with poorly fusion competent cells. Among the numerous cases of heterotypic fusion that can occur under pathological conditions, we will only discuss here the fusion of myeloid-derived cells with HIV-1-infected T lymphocytes. It is important to notice that HIV-1 infection does not trigger efficient homotypic fusion between CD4 T cells despite the fact that they express the viral fusogenic protein gp120 [11, 67, 74].

In the context of HIV-1, MGCs can arise from infection by cell-free particles (see section above) but also from the initial heterotypic fusion between infected T lymphocytes and macrophages, followed by subsequent fusions with surrounding uninfected macrophages [12]. This happens for macrophages, OCs, and dendritic cells, but not for monocytes [31, 74–76]. Interestingly, dendritic cell maturation induced by lipopolysaccharide (LPS) stimulation inhibits this fusion process [74], suggesting that activation and polarization of myeloid cells could modulate their fusogenic capacities. Although not formally detected in vivo, these heterotypic fusion events are thought to play a crucial role in the formation of infected MGCs, especially in tissues where myeloid and T cells are abundant, such as secondary lymphoid organs [12, 31, 62]. Consistent with this hypothesis, in vivo studies have shown that lymphoid tissue-resident macrophages of Simian Immunodeficiency Virus (SIV)-infected macaques contain T-cell markers and viral nucleic acids originating from infected T cells [77, 78]. The lack of formal evidence for the existence of these HIV-induced heterokaryons in vivo could be explained by the fact that, in vitro, these lymphocyte-monocyte cells retain a myeloid phenotype and rapidly downregulate T cell markers [79]. The opposite mechanism (*i.e.* fusion of infected myeloid cells with uninfected T cells) has also been proposed [80], but recent studies suggest that myeloid cells mainly transmit HIV-1 to target T cells through the formation of transient virological synapses without cell-to-cell fusion [81–83]. Many questions remain unanswered regarding the future of the lymphocyte/macrophage heterokaryons, such as the persistence and functionality of lymphocyte-derived nuclei.

## Molecular actors involved in cell-to-cell fusion

**Fig. 1:**
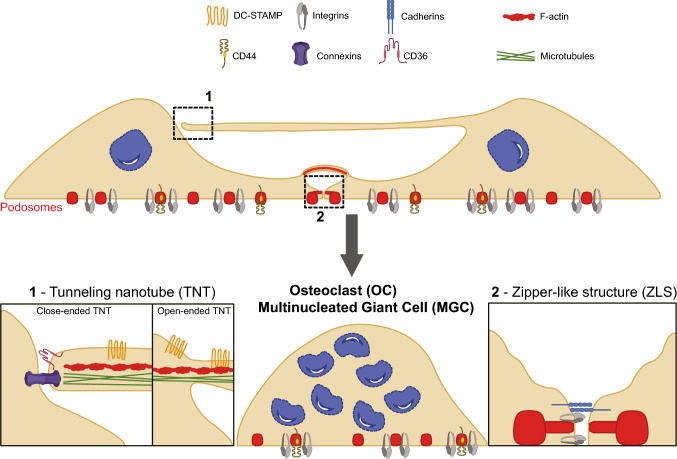
Schematics representing F-actin cellular structures and potential localization of fusion mediators on these structures during the formation of multinucleated giant cells (MGCs). In this model, the main F-actin-based structures involved in the fusion process of myeloid cells are presented: tunneling nanotubes (TNTs, insert 1) likely participate in the early stages of cell-to-cell fusion, while zipper-like structures (insert 2) stabilize adhesion between multinucleated cells in the late stages, all these structures leading to MGC formation. For more clarity, we have indicated on the figure only the proteins involved in fusion localized at these structures, the identity and the role of the other proteins being detailed in Table 1. In addition to the transport of proteins (i.e. proteins involved in the fusion process such as DC-STAMP) between OC precursors, TNTs could either be closed-ended TNTs where gap junction proteins (*i.e.* Connexin-43) and CD36 localize, or thick open-ended TNTs containing both F-actin and microtubules and aiming to mix the cytoplasm of the two cell partners. Podosomes are F-actin adherent structures present in mononucleated macrophages and MGCs. Integrins and CD44 are involved in MGC fusion and localize to the peripheral ring and to the actin core of podosomes, respectively. Podosomes could evolve into zipper-like structures (insert 2) in which adhesion proteins such as integrins and cadherins would favor strong junctions between MGCs

Some of the molecular actors involved in the mechanisms of myeloid cell fusion have been identified in OCs. Here we will focus on the cell surface proteins (see Table 1 and Fig. 1) that are involved in OC fusion. We will discuss their implications for cell-to-cell adhesion prerequisite for fusion, and we will extend their role to other types of myeloid cell fusion, mainly the formation of FBGCs. Indeed, most of the actors described in this paragraph are “helper” cell surface proteins required to initiate the fusion process, essentially involved in recognition, rapprochement or adhesion of the two cell partners. The only factors clearly described as fusogenic proteins inducing the merging of the two membranes are the syncytins, that will be discussed at the end of this paragraph.

**Table 1:** Molecular actors involved in myeloid cell fusion and their localization

Protein	Role in myeloid cell fusion	Localization to F-actin structures	References
DC-STAMP	Involved in OC and FBGC fusion in vitro and in vivo	Localizes on TNT between OC precursors	[96, 99, 100, 107]
OC-STAMP	Involved in OC and FBGC fusion in vitro Bone defects in KO mice in periodontitis model	n.d	[33, 109, 110]
Siglec-15	Involved in OC fusion in vitro and in vivo	n.d	[114–116]
Integrins	Involved in OC and FBGC fusion in vitro Essential for bone homeostasis in physiological conditions Bone defects in KO mice under pathological conditions	β2 and β3 localize in OC Zipper-Like-Structures	[124–137]
CD44	Involved in OC formation in vitro Non essential for bone homeostasis in physiological conditions Bone phenotype in KO mice under pathological conditions	Localizes to podosomes cores, especially in OC	[127, 140–142]
SIRPα	Involved in macrophage and OC fusion in vitro and in vivo	Localizes in FBGC Zipper-Like-Structures	[146–156]
CD47	Ligand of SIRPα	n.d	[145, 146, 149, 154, 157, 158]
CD36	Involved in FBGC fusion Role in OC fusion unclear	Localizes to lamellipodia and cell contact zones in FBGC	[152, 157, 159]
Tetraspanins	CD9 and CD81 inhibit MGC formation in vitro and in vivo Role in OC fusion unclear	n.d	[162–165]
E-cadherin	Involved in OC and FBGC fusion	Accumulates and form complexes with catenins at sites of cell contact in FBGC Zipper-Like-Structures	[42, 169–171]
Connexins			[173–180]
Cx-43	Involved in OC fusion in vitro In vivo bone phenotype unclear	Localizes in gap junctions between FBGC Localizes at the tip of TNT between HIV-infected macrophages	
Cx37	Involved in OC fusion in vitro and in vivo		
Syncytins	Drive the fusion of plasma membranes lipid bilayers Involved in fusion of OC and FBGC (early stages) in vitro	Localize at podosomes and filopodia in OC Concentrate at sites of cell contacts between fusing OC	[13, 22, 34]

*Cx* connexin; *CD* cluster of differentiation; *DC-STAMP* dendritic cell-specific transmembrane protein; *FBGC* foreign-body giant cell; *HIV* human immunodeficiency virus; *IL* interleukin; *KO* knock-out; *MGC* multinucleated giant cell; *n.d.* not defined; *OC* osteoclast; *OC-STAMP* osteoclast stimulatory transmembrane protein; *Siglec-15* sialic acid binding Ig-like lectin 15; *SIRPα* signal regulatory protein alpha; *TNT* tunneling nanotubes; *ZLS* zipper-like structures

### Master cell surface regulators involved in OC fusion

One of the master regulators of osteoclastogenesis is Dendritic Cell Stimulatory Transmembrane Protein (DC-STAMP) [84]. First identified as a dendritic cell surface protein, it is shared by other cells of the monocytic lineage, including OCs [85, 86]. Knock-down of this molecule abrogates FBGC and OC fusion both in vitro and in vivo, and DC-STAMP-deficient mice manifest a mild osteopetrotic phenotype associated with a lack of multinucleated OCs [84, 87, 88]. The DC-STAMP ligands are still unknown. It has been shown that the immunoreceptor tyrosine-based inhibitory motif (ITIM) on the cytoplasmic tail of DC-STAMP controls osteoclastogenesis by triggering a signaling pathway through the NFATc1/Ca^2+^ axis [89]. Moreover, DC-STAMP expression is under the influence of the RANKL/NFATc1 and STAT6/STAT-1 axes, in OCs and FBGCs respectively [35, 90–92]. Although some of the results need further investigation, and will likely depend on the experimental model used, in freshly isolated human monocytes, DC-STAMP^high^ cells seem to be the primary precursors of OCs. Surprisingly, the surface expression of this molecule decreases during the early stages of OC differentiation [89, 93, 94]. Moreover, Hobolt-Pedersen et al*.* correlated the heterogenous expression and cell surface localization of DC-STAMP along with CD47 and syncytin-1 (see below for the role of these molecules in myeloid cell fusion) with the selection of the cell partner and frequency of the fusion process [25]. While a possible transport of DC-STAMP by TNTs has been proposed [95] and will be discussed later (see “Role of podosomes and zipper-like structures in myeloid cell fusion”), the underlying mechanism linking DC-STAMP and cell-to-cell fusion remain unclear.

OC stimulatory transmembrane protein (OC-STAMP) shows similarities with DC-STAMP [35, 96]. This transmembrane protein is induced during OC differentiation and is essential for the initial steps of cell-to-cell fusion and in vitro bone resorption activity [35, 97]. In contrast to DC-STAMP-KO mice, OC-STAMP-KO mice do not present any significant bone defects [35, 97]. However, in the ligature-induced periodontitis model, bone resorption is reduced in KO mice compared to *wt* [98]. OC-STAMP is also required for FBGC formation both in vitro and in vivo [35, 99] and is under the control of the STAT6/STAT1 signaling axis [91]. It is noticeable that although both proteins are essential for osteoclastogenesis, they are not interchangeable but instead cooperate to promote cell-to-cell fusion [35, 100]. Moreover, both OC- and DC-STAMP-deficient cells retain the ability to fuse with *wt* cells, suggesting that fusion is induced through a heterologous interaction between a founder and a fusion-competent cell. This new concept of heterogeneity in fusion competence should be considered in the context of heterologous fusion between a macrophage and another cell.

Another cell-surface receptor involved in OC differentiation is the sialic acid-binding Ig-like lectin 15 (Siglec-15). Siglecs are a distinct group of the immunoglobulin superfamily that have evolved to use sialylated glycans as their predominant ligands. They are involved in the regulation of several immune cells in numerous pathologies including infectious diseases, inflammation, and cancer [101]. Siglec-15 has been first described in macrophages and dendritic cells, but it is most strongly expressed in OCs and their precursors [102]. Mice lacking Siglec-15 show mild osteopetrosis and impaired OC differentiation, and Siglec-15 antibodies reduce the fusion of murine OCs in vitro [102–104]. This function of Siglec-15 has been related to its intracellular association with the adaptor DAP12, a master actor of macrophage and OC fusion [105, 106], and Syk-dependent signaling that has been proposed to activate the RANK pathway [103]. The transcription factor(s) involved in Siglec-15-mediated enhancement of osteoclastogenesis are not known. Siglec-15 and other Siglecs have specific extracellular domains that can interact with a huge variety of ligands both in *cis* and in *trans*, thus facilitating cell-to-cell interactions [107]. One of the Siglec-15 ligands is CD44 [108], and Siglec-15 expressed at the surface of OC precursors could recognize CD44 on adjacent OC precursors to either trigger downstream signaling via DAP12 [109] or simply help to bring membranes together. Recently, Siglec-15, as well as Siglec-1/CD169, have been associated with pulmonary tuberculosis [110, 111]. The presence of these Siglecs, along with other Siglec proteins, at the surface of lung macrophages could also participate in the formation of MGCs in *Mtb-*induced granulomas.

### Adhesion receptors

Integrins mediate cell-to-extracellular matrix and cell-to-cell adhesion [112], hence they are potentially engaged in all steps of MGC formation. They are ubiquitous heterodimeric receptors composed of one α- and one β-subunits. They constitute a family of 24 members with specific tissue distribution and distinct ligand binding capacities. Expressed at the cell surface, integrins adopt a high-affinity state for ligand binding in processes defined as inside-out and outside-in signaling, and through their intracellular domains, they regulate actin cytoskeleton polymerization, among other pathways. In myeloid cells, the main adhesive structure is the podosome, which sticks to the extracellular matrix thanks to integrins and other adhesion receptors (see “Role of podosomes and zipper-like structures in myeloid cell fusion”).

The best-characterized integrin expressed by OCs is αVβ3, which binds to a variety of extracellular matrix proteins such as vitronectin, osteopontin, and bone sialoprotein [112]. In vitro differentiated OCs from β3 null mice had disorganized sealing zones and less nuclei. However, β3^−/−^ mice develop an osteosclerotic phenotype with increased numbers of OCs. The bone phenotype of deficient mice is probably due to the impaired bone resorption activity of OCs [113], along with defects in the associated signaling pathways mediated by c-Src, Syk, and DAP12 [114]. β3 integrin localizes in the diffuse actin cloud of the podosome structure and could participate in OC fusion along with another adhesion protein, CD44, which localizes to the podosome actin core [115]. In mammalian OCs, in addition to αVβ3, several other integrins are expressed, such as integrins β1, β2, and β5. Different studies have highlighted the role of integrins α9β1, αMβ2, αLβ2, as well as αVβ5 in OC fusion and bone resorption activity in vitro. However, integrin heterogeneity and potential compensation render in vivo phenotypes of integrin KO mice difficult to interpret [116–120].

The activation of integrins depends on the binding of adapter proteins, namely Kindlin-3, Talin1 and Rap1; the role of these integrin regulators in osteoclastogenesis has been assessed using elegant mouse models developed by the teams of Moser, Ginsberg and Teitelbaum [121, 122]. Using either single, double, or triple integrin β1, β2, β3-deficient OCs cultured in vitro, they first proved that only a double or triple integrin KO impaired podosome and sealing zone organization, suggesting compensatory functions between integrins. Interestingly, the phenotype of triple integrin KO OCs presents the same characteristics as kindlin-3^−/−^ OCs, specifically multinucleation is strongly impaired together with important defects in podosome organization. Moreover, kindlin-3 KO mice developed severe osteopetrosis, stronger than those of mice lacking the three integrins. Similar results were obtained with Talin1 and Rap1 KO mice [122]. Finally, the role of both β1 and β2 has also been studied in FBGCs by McNally and Anderson. Using anti-integrin β1 and β2 antibodies, they showed inhibition of FBGC adhesion and fusion [123]. Additionally, the ligand-receptor pair LFA-1/ICAM-1 was suggested to play a role in the fusion of MGCs in human blood monocytes cultured with cytokines IL2, IL-4, or TNF α [124], and in rat microglia cultured in vitro with IL-3, IL-4, gamma-INF, and GM-CSF [125].

In conclusion, although some of the results depend on the experimental model, integrins clearly play an important role in cell–matrix and cell-to-cell adhesion as well as cytoskeletal rearrangement during MGC formation. However, it is still difficult to clarify which sets of integrins control each of the stages of MGC fusion and function.

CD44 is a ubiquitous cell surface adhesion molecule involved in both cell–matrix and cell-to-cell interactions. Expressed in many cell types, including myeloid and lymphoid cells, this transmembrane protein recognizes and binds to numerous components of the extracellular matrix such as hyaluronic acid, collagens, osteopontin, and laminins [126]. CD44 is transiently induced in macrophages under fusogenic conditions, and CD44 ligands prevent multinucleation, suggesting that CD44 and its putative ligands participate in adhesion/fusion mechanisms [127]. However, the impact of CD44 deficiency on the formation of OCs remains unclear [128, 129]**.** While CD44 specific-antibodies inhibit fusion in primary bone marrow-derived OCs [130], no bone defects were observed in vivo for CD44 KO mice under physiological conditions, suggesting that in these conditions, compensating signals may exist for the loss of CD44 [128]. However, CD44 deficiency does suppress cortical bone defects induced by hindlimb unloading [129]. Thus, in vitro, CD44 appears to be involved in OC formation but its role in OC differentiation and function in vivo seems to be only revealed in some induced-bone loss models and could be strongly dependent on the pathological context.

Cadherins are transmembrane glycoproteins that mediate Ca^2+^-dependent cell-to-cell adhesion. E-cadherin is the best-characterized component of cell junctions, which contributes to the maintenance of the epithelial barrier integrity through homotypic interactions [131]. E-cadherin is also expressed in the monocyte/macrophage lineage. It is induced in a STAT-6 dependent manner consequently to IL-4 or IL-13 treatments [44, 105, 132, 133]. In IL-4 stimulated macrophages, E-cadherin forms complexes with catenins that accumulate at the sites of cell contact [133, 134]. Different approaches using treatment with specific antibodies and E-cadherin-deficient macrophages show that this protein participates through homotypic interaction in IL-4-induced MGC formation [44, 133]. E-cadherin is also involved in the OC fusion process, as blocking E-cadherin in 1,25 dihydroxyvitamin D_3_- stimulated bone marrow cells significantly reduces both the number of multinucleated OCs and bone resorption activity [135]. This effect was confirmed in RANKL-treated RAW 264.7 macrophage cells, in which inhibition of E-cadherin impairs OC fusion and delays early stages of osteoclastogenesis [136]. It is also interesting to note that E-cadherin could be involved in heterotypic interactions and, potentially, heterotypic fusion [133], in particular between macrophages and T cells.

Connexins are a multigene family of hemichannel- and gap junction-forming proteins. Connexin-43 (Cx43) is the major connexin protein expressed in developing and mature skeletal tissues. This protein is abundant in OCs and OC precursors in mouse and human [25, 137–139]. So far, little is known about the precise function of gap-junction proteins in OC formation, but blocking gap-junctional communication and particularly Cx43 inhibits OC fusion and bone resorption in vitro [137–139]. In mice, Cx43 is required for both skeletal development and maintenance, particularly in cortical bone. However, because Cx43 is expressed and functional in a wide variety of bone cells, including OCs, osteoblasts and osteocytes, these skeletal phenotypes remain difficult to interpret [140, 141]. While Cx43 involvement in OC fusion has been proposed but not fully understood [141], some studies also proposed a role for gap junctions in the fusion of myeloid cells (FBGCs) based on immunohistochemistry and ultrastructural immunogold labelling showing that Cx43 localizes between fusing macrophages [142, 143]. In addition, with a lower expression level in OCs compared to Cx43, Cx37 has also been recently described to participate in OC fusion and differentiation both in vitro and in vivo, with higher bone density in Cx37 KO mice compared to controls [144]. Thus, further studies are needed to better understand the role of the different connexins in MGC formation.

### Other cell-surface receptors

The signal regulatory protein α (SIRPα) also known as MFR (Macrophage fusion receptor) was one of the first molecules implicated in macrophage fusion. This transmembrane glycoprotein of the superfamily of immunoglobulins possesses a cytoplasmic tail containing multiple ITIMs and is highly expressed in myeloid cells. Its interaction with the integrin-associated transmembrane protein CD47 is essential for adhesion leading to macrophage multinucleation and to OC fusion both in vitro and in vivo [145–150]. Moreover, during osteoclastogenesis, disruption of the CD47-SIRPα association leads to a lack of SIRPα phosphorylation, a defect in Src homology 2 domain-containing protein tyrosine phosphatase (SHP) recruitment, and impaired dephosphorylation (i.e., impaired inhibition) of the non-muscle actin-based motor myosin IIA (MyoIIA) [151]. This is consistent with the transient decrease of MyoIIA expression required to trigger OC fusion [152]. It is important to note that in addition to its interaction in *trans* with SIRPα, CD47 can also bind in *cis* a set of integrins and the extracellular matrix glycoprotein, thrombospondin-1 (TSP-1) [153]. For example, in concert with the cell-surface protein CD36 (see below), CD47 also participates in TSP1-mediated OC formation [154]. In addition to this complexity, Podolnikova et al. recently reported that the macrophage integrin 1 antigen (Mac-1) also interacts in *cis* with CD47, becoming another ligand of SIRPα in *trans*, and that their interaction could be involved in macrophage fusion [155]. In contrast to the myeloid restricted expression of SIRPα, CD47 is widely and variably expressed in all types of cells. Its interaction with SIRPα transmits an anti-phagocytic signal to macrophages, known as the "don't eat me" signal that protects cells from macrophage engulfment. In many cancer cells and in immune cells during infection, CD47 is upregulated, allowing the cells to evade innate immune detection [156–158]. If we consider the active role of the SIRPα/CD47 axis in the fusion process between two myeloid cells, we assume that this interaction triggers two mechanisms: first, it inhibits phagocytosis of the target cell by macrophages; and second, it promotes the fusion of this target cell with macrophages, giving rise to heterotypic or homotypic giant cells. Hence, regulating the expression of SIRPα/CD47 might be of pivotal importance to control MGC formation and survival.

Using an unbiased antibody screening strategy to identify mediators of macrophage fusion induced by IL-4, Helming et *al.* identified the class B scavenger receptor CD36 [159]. CD36 (also known as platelet glycoprotein 4) is a membrane receptor with an extensively glycosylated extracellular domain flanked by two transmembrane domains. It is expressed in a variety of cell types and binds a diverse array of ligands, including oxidized low-density lipoproteins, non-opsonized bacteria, and ligands on apoptotic cells. As a molecule specialized in sensing and internalizing lipids, the role of CD36 at the surface of macrophages has been mainly described to mediate foam cell formation in atherosclerosis [160]. In the context of MGC formation, CD36 is necessary for IL-4-mediated murine macrophage fusion in vitro via the recognition of phosphatidylserine exposed at the membrane of the target cell before membrane merging [159]. The role of this protein in OC fusion is not obvious [154, 159], but CD36 could cooperate with CD47 as mentioned before [154]. It is likely that the role of CD36 in OC fusion could also involve its interaction with phosphatidylserine on OC precursors, as osteoclastogenesis has been shown to be controlled by the phosphatidylserine-regulated activity of several proteins in an in vitro model of synchronized fusion of OC precursors [161].

Tetraspanins constitute a large family of four-pass transmembrane proteins, which are ubiquitously expressed. Through their association with numerous partners, including integrins, cytoskeletal proteins, and signaling molecules, tetraspanins organize specialized membrane microdomains (tetraspanin-enriched microdomains) and participate in many biological processes including cell adhesion, migration, and different types of cell-to-cell fusion [162]. Tetraspanins, such as CD9 and CD81, are required for muscle- and sperm-egg fusion mechanisms [163]. In contrast, when stimulated in vitro or in vivo, alveolar macrophages and bone marrow cells of CD9- and CD81-KO mice form more MGCs compared to *wt,* and double-null mice spontaneously develop MGCs in the lung [164]. This inhibitory role of CD9 and CD81 in phagocyte fusion has also been supported in an experimental system using Concanavalin A-induced fusion, while in this model, another tetraspanin, CD63, promoted MGC formation [164–166]. The role of tetraspanins in OC fusion is more controversial. Bone marrow cells from CD9-null mice stimulated in vitro display enhanced OC fusion, and CD9 and CD81 double null mice show increased OC number associated with a loss in bone mineral density [164]. In contrast, CD9 inhibition by siRNA or blocking antibodies reduces fusion in vitro [167]. More recently, other tetraspanins, such as CD82, have also been reported to regulate OC fusion [168]. Thus, due to their partner multiplicity, their heterogeneity, and their involvement in numerous biological functions beyond cell-to-cell fusion, the roles of tetraspanins in myeloid cells still remain elusive.

### Fusogenic proteins

Fusogenic proteins induce the merging of two lipid membranes. Today, syncytins are the only fusogenic proteins implicated in the fusion of myeloid cells. They are composed of syncytin-1 and -2 in humans [169], and syncytin-A and -B in mice [170, 171]. Although the murine and human syncytin genes are not orthologous, they are all derived from retroviral genes that have been stably integrated in the mammalian genome [172]. These viral envelope proteins are involved in several physiological processes of cell-to-cell fusion such as the formation of the syncytiotrophoblasts [169] or the fusion of myoblasts into myotubes [173], upon interaction with ASCT-2 [169]. The role of syncytins in OC multinucleation has been investigated in both humans and mice. In humans, syncytin-1 is transiently expressed at early stages of OC differentiation [24, 36, 174], whereas the expression of its receptor ASCT-2 is enhanced at early steps and remains stable [36]. Inhibition of syncytin-1 blocks fusion of OCs in the early stages with no impact on OC number [13, 36]. Furthermore, this inhibition has no effect on bone degradation activity by mature OCs [36]. Interestingly, in human OCs, syncytin-1 co-localizes with F-actin in podosomes and filopodia and then concentrates in the contact zone when the two partners are in close proximity [36]. In mice, a comparison between syncytin B^−/−^ and *wt* OCs differentiated ex vivo shows that syncytin B plays a role in the fusion of OCs at initial stages but it does not seem essential for bone resorption activity. In agreement with these in vitro observations, syncytin-B-deficient mice show a normal bone phenotype [24]. The effect of syncytin in myeloid cell fusion is not limited to OCs since there is an inhibition in the number of IL4-derived multinucleated cells from the bone marrow of syncytin-B deficient mice compared to *wt*. However, these mice did not show any change in the number of FBGCs formed in response to implanted foreign material [24]. The role of syncytins in the fusion of myeloid cells in different contexts is consistent with the fact that syncytin expression is not dependent on RANKL signaling [36] and thus not restricted to OCs. Although it is clear that syncytins are involved in several contexts of myeloid cell fusion, further work is needed to reconcile the different observations. Importantly, it appears that altering syncytin function in OCs inhibits their capacity to fuse but not to degrade bone matrices, suggesting that these two criteria may not be systematically dependent on each other [13].

## Role of podosomes and zipper-like structures in myeloid cell fusion

Cellular fusion is a multistage process, and each step appears to rely on the actin cytoskeleton. Live-cell imaging has uncovered a variety of actin-based structures between fusion partners but their precise roles remain to be identified. Podosomes, particularly prominent in cells of the monocytic lineage such as macrophages, dendritic cells, and OCs, are multifunctional F-actin structures that combine several key abilities required in particular for cell migration and invasion [175–177]. The podosome function repertoire includes well-established functions such as cell-substrate adhesion, degradation of the extracellular matrix, and rigidity and topography sensing [176, 177], but were also proposed to be involved in cell protrusion stabilization, cell migration in 3D environments and cell-to-cell fusion [176, 178–180]. Podosomes present a submicron-size core of Arp2/3-mediated branched F-actin and actin-regulatory proteins, including WASP and cortactin. This core is surrounded by an adhesion ring comprising integrins and proteins linking integrins to the actin cytoskeleton, such as vinculin and talin. Podosomes are dynamic entities forming the basis of different structures depending on cell types and differentiation stages. In OCs, podosomes are collectively and sequentially organized into different high-ordered structures along the lifespan and activity of the cells and the properties of the matrix: clusters, rings, podosome belts, and sealing zones [16, 29, 101, 180, 181], the latter being the functional structure confining bone resorption [182].

Zipper-like structures, which are also podosome-related structures, have been described recently at the initial stages of cell-to-cell adhesion in keratinocytes [183] and during the fusion of myoblasts into myotubes [1]. Their name comes from the periodic actin distribution that resembles a zipper. In contrast to other actin structures present in individual cells and responsible for cell–matrix interactions, the zipper-like structures are involved in cell-to-cell interactions. This transient structure exhibits the unique ability to bridge two plasma membranes. The present paragraph discusses the role of podosomes or podosome-like structures in the process of myeloid cell fusion, particularly during osteoclastogenesis and IL-4 induced MGCs (FBGC formation).

Two studies first revealed the presence and the role of podosome-like structures in the fusion of OCs [184, 185]. Zipper-like structures were observed during OC fusion in the murine macrophage cell line RAW 264.7 and in OCs derived from mouse bone marrow macrophages. It is important to note that complete OC differentiation is the result of a combination of fusions involving heterotypic precursors with sometimes different numbers of nuclei [29]. In addition to podosome-like structures observed at early steps of OC fusion that could be the origin of the podosome belts in mature OCs, later stages of fusion (*e.g.,* fusion of multinucleated cells) involve canonical zipper-like structures formed at the cell contact sites followed by fusion of the podosome belts and finally apposition of the plasma membrane [185, 186]. The actin flow in the zipper-like structure is proposed to generate forces that facilitate adhesion between multinucleated partner cells. In addition, in the center of these structures, the plasma membranes of the two partner cells form close contact sites where membrane fusion would be promoted [29, 185, 186]. Importantly, zipper-like structures have also been observed in vitro*,* in OCs derived from chicken bone marrow cells [187] and in vivo in OC found in mouse calvariae [185]**.**

Several proteins of podosomes are present in zipper-like structures of OCs. For example, Arp2/3 and cortactin colocalize with actin at the center of the zipper-like structures, whereas integrin β3, paxillin, vinculin, and zyxin localize to the periphery of the structure [185, 188]. As for podosome formation in primary macrophages, zipper-like structure formation is dependent on Tks5, a Src substrate [189], and its stability is affected by Arp2/3 inhibition [184, 188]. In addition, MyoIIA-mediated actin contractility seems to be an inhibitory checkpoint for cell fusion in several contexts [1], including OC multinucleation [152]. However, inhibition of myosin light chain kinase with ML-7 has shown little effect on zipper-like structure organization [188], suggesting that zipper-like structure formation is rather mediated by branched actin elongation than by actomyosin contraction.

To our knowledge, the involvement of the molecular actors of cell-to-cell fusion found in podosomes (CD44, E-cadherin, integrins; see “Different types of multinucleated giant cells from the monocytic lineage” and Table 1) in zipper-like structure formation or stability has not been examined. Of note, most of the studies describing the role of podosome-like structures in OC fusion have been performed in mouse mainly using the RAW 264.7 cell line. It could be important to decipher the mechanisms of zipper-like structure formation and dynamics in more physiological OC models.

A recent and elegant study demonstrated the existence of zipper-like structures in FBGCs. Indeed, this study revealed zipper-like structures that arise between mouse MGCs induced by IL-4 stimulation in vitro and following biomaterial implantation in vivo [134]. Using live imaging and super-resolution microscopy, the group of Ugarova showed that podosomes are the precursors of these zipper-like structures. As a consequence, and similarly to OC zipper-like structures described above, these zipper-like structures contain many podosome proteins, and their assembly and stability are dependent on Arp2/3, Wasp, and Cdc42. Furthermore, they found that the junctional protein E-cadherin and SIRPα localize at the intercellular space between adjacent cell membranes within zipper-like structures[134]. Interestingly, these two proteins have been linked to macrophage fusion (see “Molecular actors involved in cell-to-cell fusion”). From these data, it can be proposed that these structures are not involved in early cell fusions of IL-4-derived MGCs because zipper-like structures appeared at late stages of the fusion process. However, it is likely that they stabilize the adhesion of multinucleated cells, suppressing migration and/or inducing proteolysis, and thus participating in the later stages of fusion.

Although zipper-like structures described in OCs and in FBGCs share similar features in terms of morphology, dynamics, and association with podosome components, they may have different functions and thus require further analysis.

## Tunneling nanotubes and myeloid cell fusion

In addition to zipper-like structures, alternative structures, including filopodium-like protrusions, may also participate in the fusion process [29, 186, 190]. These structures show a morphological similarity to so-called TNTs [191–193]. For the past 15 years, TNTs have received scientific attention as a type of intercellular communication machinery*.* They form particularly between myeloid cells (macrophages, OCs, and dendritic cells). The identification of TNTs was a crucial turning point in the research field of intercellular communication as they possess the capacity to transfer cytoplasmic molecules, proteins, organelles and even pathogens between cells. This ability constitutes the main functional criterion for TNT definition. Some TNTs are closed, that could form a gap junction between the tip of the protrusion and the targeted cell (close-ended TNTs) [194]. Alternatively, closed TNTs could be immature ones before they fuse with the acceptor cell (open-ended TNTs). Here, for simplicity reasons, we will use the word TNTs for both closed- and open-ended structures.

In macrophages, TNTs have been classified into two types with different functions: thin TNTs (diameter of less than 5 µm), containing only F-actin; and thick TNTs, which contain both actin and microtubules (diameter ranging from 5 to 20 µm) [111, 195–197]. Only thick TNTs are able to transport large organelles such as lysosomes, mitochondria, and even, albeit questionably, nuclei [198].

During human or murine OC fusion, it has been observed that OC precursors form abundant TNTs prior to cell-to-cell fusion [31, 195, 199, 200] that resemble the thick and thin TNTs described in macrophages. A role of TNTs in the fusion process has been suggested [195, 199, 200], potentially to help in fusion partner rapprochement. M-Sec has been identified as a key factor in macrophage TNT formation [195, 201, 202]. Consistent with this, the expression level of M-Sec increases during osteoclastogenesis, and M-Sec depletion significantly suppresses OC differentiation and fusion [95, 199]. Furthermore, there is now evidence of intercellular transport of DC-STAMP through TNTs formed between OC precursors [95]. In addition, large TNTs may provide a route for nuclei transport during OC fusion [198].

Myosin X (MyoX) is a molecular motor that utilizes ATP to perform many cellular functions, including TNT formation and elongation between neuronal cells [203]. This unconventional myosin is also expressed in OCs [200]. Mice with MyoX loss of function exhibit osteoporotic defects, which are likely due to increased osteoclastogenesis and bone resorption as bone formation markers were unchanged [204]. This in vivo phenotype is in contradiction with in vitro observations. Actually, in addition to defects in F-actin organization (*i.e.,* sealing zone formation) and adhesion of mature OCs, OC precursors with reduced levels of MyoX expression by an shRNA approach remained mononucleated and unable to fuse. These results were correlated with a significant decrease in TNT formation in the absence of MyoX. Surprisingly, this effect is independent of M-sec expression, suggesting another potential mechanism for TNT-mediated OC fusion [200]. In addition, the presence of heterotypic TNTs between endothelial progenitor cells and OC precursors boosts OC fusion. Although these structures do not induce heterotypic fusion, they could indirectly permit OC precursors to acquire fusogenic capacity [205].

Despite a lack of functional assays, filopodium-like protrusions have been described during the fusion of FBGCs in vitro [190]. Long intercellular F-actin structures (up to a few hundred microns) could be a way for a fusion-competent cell to identify a distant fusion partner. In addition, once the two cells are in close proximity, thin and short actin protrusions (around ten microns) emerging from the leading-edge of the cells are observed [190]. Interestingly, protrusions originate from sites that are enriched in podosomes which could play a role in the stabilization of cell-to-cell adhesion and, more hypothetically, in the fusion of the two inter-connected cells. These two types of actin protrusions could be the TNT-like structures observed during OC fusion [29, 185].

Another potential role for TNTs in cell-to-cell fusion concerns HIV-1 infection of macrophages [111, 197, 206, 207], which are prone to form MGCs (see “Introduction”). In this context, we revealed a correlation between the formation of TNTs at the early stages of HIV-1 infection of human macrophages and the extent of the fusion later on [57, 111, 195, 197, 208]. In terms of molecular actors, M-Sec mediates rapid and efficient cell-to-cell transmission of HIV-1 at an early phase of infection by enhancing both cell motility, TNT formation, and number of nuclei per infected macrophages [207]. Finally, Cx43 localizes at the tip of close-ended TNTs formed between HIV-infected macrophages, suggesting that stabilization of long-range gap junction-dependent communication could favor HIV-1 transfer [206]. In this context, the specific role of TNTs in macrophage fusion has not been examined, nor the relevance of closed- and open-ended TNTs in pathogen spread [195]. HIV-1 transfer from infected CD4 T cells to macrophages is also mediated by a fusion mechanism, however, whether heterotypic TNTs form during this process is not known.

Despite remarkable advances in TNT biology over the last decade, formidable challenges in this discipline remain, including TNT function, heterogeneity, and existence in vivo. TNT structures seem much more versatile than zipper-like structures. They are morphologically heterogeneous and expressed at early but also at late stages of macrophage fusion. It is clear that TNTs mediate continuity between remote cells for cargo transport, and thus could transport proteins or signaling molecules involved in cell-to-cell fusion. In addition, it is likely that TNTs and zipper-like structures exert physical forces that bring the two connected cells closer together and promote their fusion. The molecular components driving cell-to-cell fusion and the existence of TNTs in vivo also need to be examined. Many questions still remain, in particular, further investigation is required to determine the structural similarities between TNTs and zipper-like structures and the contribution of these two F-actin structures in cell-to-cell fusion process [186, 209]. Some studies in the literature suggest a differential involvement of these structures depending on the type of MGCs and/or the stage of the fusion process.

## Perspectives

The fusion of myeloid cells is a hallmark of osteoclastogenesis but it is also involved in numerous pathologies. However, the gain in the function of multinucleation is still an enigma. In the particular case of OCs, the efficiency of fusion usually correlates with bone resorption activity, and deregulation in the number of nuclei leads to bone diseases, such as osteopetrosis or Paget's disease, suggesting that the physiological cell-to-cell fusion process must be strictly controlled [30, 31, 33, 93]. Regarding the other MGCs, they are generally associated with pathological conditions and most have exacerbated functions. Therefore, it is of interest to be able to control their formation and for that, it is necessary to better understand their fusion mechanisms. Although not universal, the molecular machinery involved in the fusion process seems to be shared, at least partially, between OCs and MGCs in different contexts (Fig. 1). However, some specificities exist in the nature of the molecular actors and the actin-based structures involved, depending on the type of MGCs and the stage of the cell-to-cell fusion process. There are still significant gaps in the characterization of the molecular determinants required for MGC formation.

An interesting avenue of research would be to take advantage of these specificities and identify fusion molecules or structures specifically linked to discrete MGC-associated diseases. The identification of specific markers could considerably advance the diagnosis of these diseases and lead to the discovery of new drugs with a more controlled impact. Moreover, most of the fusion molecules identified so far promote or stabilize the fusion process, and only a few, *i.e.,* tetraspanins, have been identified as fusion inhibitors. It would be interesting to study whether these proteins, in a physiological context, are sufficient to limit the natural fusogenic capacity of myeloid cells and prevent macrophages from fusing with their counterparts or with other cell types. Looking for new inhibitory actors of the fusion process could also help to better understand how, under fusogenic conditions, this inhibition could be bypassed.

Another objective will be to develop robust models to study MGC formation, especially in vivo. Indeed, most of the studies have been performed in vitro*,* and in vivo approaches often fail to show similar effects. Although the in vitro models, generally limited to a single cell type cultured at high density, provide valuable information for the identification of critical actors in macrophage fusion, they could bias the choice of the F-actin structures involved. For example, at an advanced stage of OC differentiation, the proximity of MGCs will favor the formation of zipper-like structures with the emergence of fine TNT-type structures that induce the fusion process [29, 95, 134]; the presence of these structures still needs to be demonstrated on a relevant substrate, such as bone matrix for OCs, and in more complex three-dimensional settings. In addition, the existence of TNTs in vivo is still a matter of debate in the field, and has never been described in the bone environment. Finally, the quite recent concept of heterogeneity in the two fusion partners should be considered in future investigations of new actors in MGC formation.

In conclusion, important gaps still exist in our understanding of the mechanisms of myeloid cell fusion. The identification of specific actors in MGC formation will be important for identifying either markers or potential targets for MGC-associated pathologies.

## Data Availability

Not applicable.
